# Antagonist muscle torque at the ankle interfere with maximal voluntary contraction under isometric and anisometric conditions

**DOI:** 10.1038/s41598-022-24752-y

**Published:** 2022-11-24

**Authors:** Maxime Billot, Julien Duclay, Philippe Rigoard, Romain David, Alain Martin

**Affiliations:** 1 PRISMATICS Lab (Predictive Research in Spine/Neuromodulation Management and Thoracic Innovation/Cardiac Surgery), Poitiers University Hospital, 2 Rue de La Milétrie, Poitiers, France; 2 Toulouse NeuroImaging Center, Université de Toulouse, Inserm, UPS, Toulouse, France; 3 Department of Spine Surgery and Neuromodulation, Poitiers University Hospital, 86021 Poitiers, France; 4 ISAE-ENSMA, Pprime Institute UPR 3346, CNRS, University of Poitiers, 86360 Chasseneuil-du-Poitou, France; 5 Laboratoire INSERM U1093 Cognition, Action et Plasticité Sensorimotrice, Université de Bourgogne - UFR STAPS, Dijon, France

**Keywords:** Neuroscience, Motor control

## Abstract

While resultant maximal voluntary contraction (MVC) is commonly used to assess muscular performance, the simultaneous activation of antagonist muscles may dramatically underestimate the strength of the agonist muscles. Although quantification of antagonist torque has been performed in isometric conditions, it has yet to be determined in anisometric conditions. The aim of the study was to compare the mechanical impact of antagonist torque between eccentric, isometric and concentric contractions in PF and DF MVCs. The MVCs in dorsiflexion (DF) and plantar-flexion (PF) were measured in isometric, concentric and eccentric conditions (10° s^-1^) in nine healthy men (26.1 ± 2.7 years; 1.78 ± 0.05 m; 73.4 ± 6.5 kg) through two sessions. Electromyographic (EMG) activities from the soleus, gastrocnemius medialis and lateralis, and tibialis anterior muscles were simultaneously recorded. The EMG biofeedback method was used to quantify antagonist torque. Resultant torque significantly underestimated agonist torque in DF MVC (30–65%) and to a lesser extent in PF MVC (3%). Triceps surae antagonist torque was significantly modified with muscle contraction type, showing higher antagonist torque in isometric (29 Nm) than in eccentric (23 Nm, *p* < 0.001) and concentric (14 Nm, *p* < 0.001) conditions and resulting in modification of the DF MVC torque-velocity shape. Estimation of the antagonist torque in isometric or anisometric conditions provides new relevant insights to improve neuromuscular performance assessment and to better design strength training and rehabilitation programs related to the torque applied by agonist and antagonist muscles.

## Introduction

Muscular performance is classically assessed through analysis of the mono-articular resultant torque developed during maximal voluntary contraction (MVC) under isometric or anisometric conditions. While the resultant torque assessment provides one measurement, it reflects the activation of the multiple synergist muscles around a joint^1^, thereby highlighting the coactivation phenomenon^1–5^. While it has been clearly demonstrated that antagonist muscles are electrically active during a high level of performance, antagonist assessments and their mechanical interpretation and impact on resultant torque are still unclear in the literature.

Coactivation phenomenon can be quantified by either electromyographic (EMG) activity^2,3,6^ or by their corresponding mechanical contributions^7–11^. EMG activity analysis of antagonist muscle contribution, named coactivation level, is related to the percentage of muscle activation acting as antagonist^2^, and can be interpreted as the reflection of corticospinal neural drive^7^. On the other hand, the mechanical contribution of antagonist muscle, named mechanical ratio, is related to the percentage of antagonist torque over the agonist torque^5^, and provides measurement of mechanical output. Although coactivation level and mechanical ratio could be considered as complementary indicators of motor control output, previous studies have reported that coactivation level does not necessarily reflect the mechanical ratio, depending on the muscles involved^5,7–11^, angle joint^7^, age^8,11^ and sex^9,10^. Estimation of antagonist torque based on coactivation level can result in misinterpretation of about 5–15% compared to the mechanical ratio^5,7^. More specifically, we previously demonstrated that plantar-flexor (PF) muscles acting as antagonist exerted a torque of about 30 Nm during dorsiflexion (DF) MVC, causing underestimation of ~ 70% of the maximal torque capacity of the dorsal flexors^5,7^. In addition, with a similar coactivation level, DF muscles exerted a torque of about 3–6 Nm and underestimated only ~ 3% of PF MVC^5,7^. These findings in isometric conditions are difficult to transpose to ecological movements, and extension of our knowledge to anisometric conditions remains to be carried out. Even though coactivation level apparently does not differ between muscle contraction types in anisometric conditions^12–16^, it has been clearly established that similar EMG activity results in greater torque in eccentric than in concentric sub-maximal contraction^17–20^. The only measure available in anisometric condition is the coactivation level, which does not reflect the mechanical impact of antagonist muscles.

To bridge this gap, we aimed to compare the mechanical impact of antagonist torque between eccentric, isometric and concentric contractions in PF and DF MVCs. Based on isometric studies^5,7^, we hypothesized that resultant MVC strongly underestimates agonist MVC in DF and to a lesser extent in PF. In addition, since similar EMG activity resulted in greater torque in eccentric than in concentric conditions^17–20^, we expected that antagonist torque had greater mechanical impact during concentric MVC (antagonist acting in eccentric) than in eccentric MVC (antagonist acting in concentric) conditions, resulting in a modification of the torque-velocity relationship. Our results should provide substantial clues to identify muscular deficit in high-level sport practice and rehabilitation programs. While coactivation is currently considered via EMG activity only in clinical practice, our results could help clinicians to better design rehabilitation programs by determining strengths and weaknesses in isometric and dynamic contractions taking into account the agonist/antagonist mechanical contribution.

## Results

### Reproducibility: intra-class correlation

The ICC scores are presented in Table 1 for both PF and DF MVCs. Measurements in PF MVC showed excellent reliability with ICC ranging from 0.81 to 0.90, and the TA antagonist torque in eccentric condition (0.78) and the coactivation level of the TA showed good reliability (0.71–0.75). Resultant MVC, agonist MVC, antagonist torque, and coactivation level showed excellent reliability during DF MVC, ranging from to 0.81 to 0.97.

**Table 1:** Session 1 and 2 intraday reliabilities for resultant MVC (maximal voluntary contraction), agonist MVC, antagonist torque, and co-activation level for both DF (dorsi-flexion) and PF (plantar-flexion) MVCs in three muscle contraction type conditions: eccentric, isometric and concentric.

			Session 1 versus 2 mean (SD)	ICC (95% CI)	Mean CV (SD)	Typical error
PF MVC (N m)	Resultant	Eccentric	152.1 (47.7) versus 149.4 (45.7)	0.95 (0.78–0.99)	7.5 (6.5)	7.2
		Isometric	148.8 (50.6) versus 150.3 (43.2)	0.97 (0.86–0.99)	6.4 (4.6)	6.1
		Concentric	125.8 (41.7) versus 119.7 (35.2)	0.95 (0.78–0.99)	9.0 (3.6)	7.5
	Agonist	Eccentric	153.5 (47.9) versus 150.5 (45.9)	0.95 (0.78–0.99)	7.3 (6.5)	7.5
		Isometric	151.8 (49.1) versus 154.1 (41.8)	0.96 (0.85–0.99)	6.6 (4.6)	6.4
		Concentric	127.6 (41.2) versus 121.0 (34.6)	0.94 (0.76–0.99)	9.2 (3.8)	7.7
	TA antagonist	Eccentric	1.8 (1.2) versus 1.8 (1.5)	0.90 (0.61–0.98)	35.3 (36.3)	0.3
		Isometric	3.0 (2.8) versus 3.8 (2.3)	0.86 (0.49–0.97)	33.9 (19.6)	0.7
		Concentric	1.4 (0.7) versus 1.2 (0.8)	0.78 (0.29–0.99)	29.3 (18.9)	0.2
	Coactivation level TA	Eccentric	7.2 (1.3) versus 7.6 (1.3)	0.75 (0.16–0.94)	7.5 (6.0)	0.4
		Isometric	9.8 (2.9) versus 9.9 (2.0)	0.71 (0.09–0.93)	9.3 (7.6)	0.8
		Concentric	8.0 (1.1) versus 8.4 (1.3)	0.73 (0.18–0.93)	6.5 (6.4)	0.4
DF MVC (N m)	Resultant	Eccentric	50.5 (7.3) versus 50.0 (6.6)	0.89 (0.60–0.98)	4.9 (2.5)	1.7
		Isometric	45.4 (6.5) versus 45.2 (6.1)	0.91 (0.64–0.98)	4.5 (1.9)	1.4
		Concentric	35.3 (5.2) versus 34.7 (6.3)	0.86 (0.51–0.97)	6.3 (5.3)	1.4
	Agonist	Eccentric	63.8 (11.5) versus 64.1 (9.4)1	0.90 (0.61–0.98)	4.8 (4.3)	2.1
		Isometric	76.4 (11.9) versus 72.3 (10.2)	0.97 (0.87–0.99)	4.0 (2.5)	2.2
		Concentric	59.4 (9.3) versus 55.9 (8.9)	0.81 (0.38–0.95)	7.8 (4.2)	3.1
	TS antagonist	Eccentric	24.0 (7.6) versus 21.2 (6.2)	0.84 (0.44–0.96)	15.8 (7.9)	2.4
		Isometric	31.0 (7.5) versus 27.1 (7.5)	0.90 (0.63–0.98)	12.6 (10.1)	2.3
		Concentric	14.4 (5.7) versus 14.1 (6.4)	0.84 (0.45–0.96)	17.0 (19.0)	1.3
	Coactivation level TS	Eccentric	15.2 (3.7) versus 13.4 (2.6)	0.84 (0.44–0.96)	5.8 (6.3)	1.1
		Isometric	12.8 (3.3) versus 12.3 (3.5)	0.85 (0.46–0.96)	5.3 (7.4)	0.9
		Concentric	15.7 (5.1) versus 14.2 (7.0)	0.97 (0.84–0.99)	5.8 (6.0)	0.7

*TA* tibialis anterior, *TS* triceps surae, *CV* coefficient of variation, *SD* standard deviation.

Based on ICC results, data were pooled, analyzed and presented as a mean of the 2 sessions for the following results.

### PF MVC

Resultant and agonist MVC, TA antagonist torque. The two-way ANOVA analysis showed a significant main effect of considered PF MVC presenting greater resultant than agonist MVC (*p* < 0.001, F = 29.76; d =  − 0.05 [− 0.60; 0.50]), and a main effect of muscle contraction type (eccentric vs isometric vs concentric, *p* < 0.001, F = 28.01), and a significant interaction between MVC measurements and muscle contraction type (*p* = 0.014, F = 5.60). PF resultant MVC was significantly lower than PF agonist MVC for isometric (difference between agonist and resultant MVC: 3.4 Nm, *p* < 0.001; d =  − 0.07 [− 1.07; 0.92]), concentric (difference between agonist and resultant MVC: 1.8 Nm, *p* = 0.004; d =  − 0.05 [− 1.05; 0.95]) and eccentric (difference between agonist and resultant MVC: 1.3 Nm, *p* = 0.031; d =  − 0.03 [− 1.27; 0.97]) muscle contraction type.

Regarding resultant MVC, results showed that PF concentric MVC (122.5 ± 37.8 Nm) was significantly lower than eccentric (150.7 ± 45.8 Nm, *p* < 0.001; d =  − 0.67 [− 1.70; 0.35]) and isometric MVCs (149.6 ± 46.4 Nm, *p* = 0.011; d =  − 0.64 [− 1.66; 0.39]), whereas no significant difference was found between eccentric and isometric MVCs (*p* = 0.786). Similarly, we found that PF agonist MVC in concentric MVC (124.3 ± 37.2 Nm) was significantly lower than eccentric (152.0 ± 46.0 Nm, *p* < 0.001; d =  − 0.66 [− 1.69; 0.36]) and isometric MVC (153.0 ± 45.0 Nm, *p* < 0.001; d =  − 0.69 [− 1.72; 0.34]), whereas no significant difference was found between eccentric and isometric MVCs (p = 0.830).

**Figure 1:**
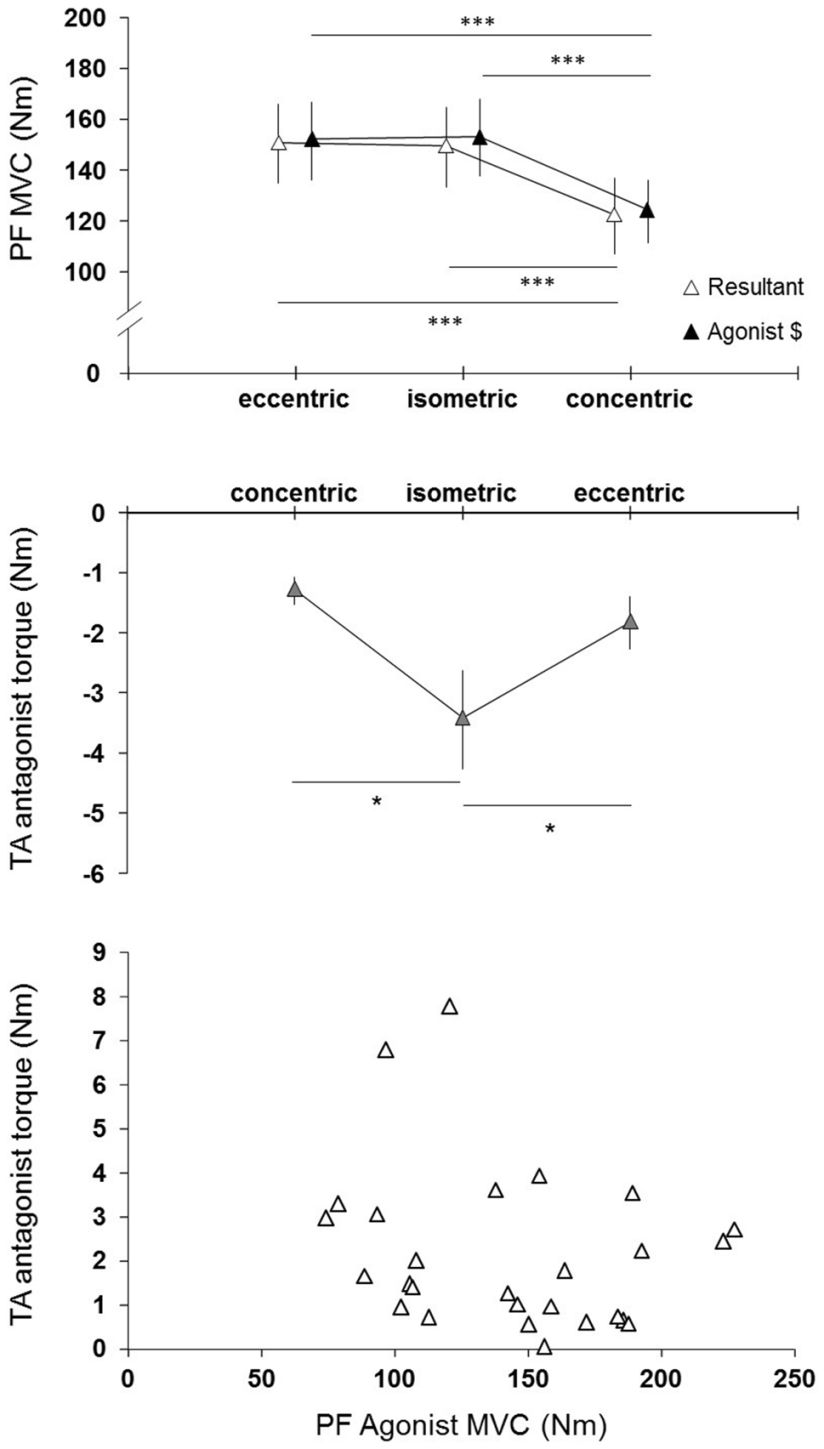
Mean PF resultant and agonist MVC (upper panel), mean TA antagonist torque (middle panel), relationship between agonist MVC and antagonist MVC in every muscle contraction type (lower panel).

Results showed a significant main effect of muscle contraction type on TA antagonist torque (*p* = 0.014, F = 5.60) (Fig. 1, middle panel). TA antagonist torque was significantly higher in isometric (3.4 ± 2.4 Nm) than in concentric MVC (1.3 ± 0.7 Nm, *p* = 0.005; d = 1.19 [0.10; 2.27]), whereas no significant difference was found between eccentric (1.8 ± 1.3 Nm) and isometric (*p* = 0.028) or concentric MVC (*p* = 0.433). In addition, no correlation was observed between PF agonist MVC and TA antagonist torque (Fig. 1, lower panel).

Eccentric/concentric ratio for resultant and agonist MVC. Statistical analysis revealed a trend to higher eccentric/concentric ratio for resultant (1.24 ± 0.13) than for agonist MVC (1.23 ± 0.12, *p* = 0.056, t = 2.23).

### TA coactivation level versus mechanical ratio

Results showed a significant main effect of muscle contraction type (*p* = 0.013, F = 5.71) and coactivation method (*p* < 0.001, F = 108.3) on coactivation quantification, whereas no significant interaction between muscle contraction type and coactivation method was found (*p* = 0.93, F = 0.07) (Table 2). Post-hoc analysis showed that coactivation methods were significantly higher in isometric than in concentric (*p* = 0.004, t = 3.34; d = 0.89 [− 0.15; 1.95]), while no significant difference was found between isometric and eccentric (*p* = 0.054, t = 2.11), and between eccentric and concentric contractions (*p* = 0.24, t = 1.23). In addition, coactivation level was significantly higher than mechanical ratio (*p* < 0.001, t = 10.41; d = 2.19 [1.50; 2.88]) regardless of muscle contraction type.

**Table 2:** Coactivation level and mechanical ratio for both TA (tibialis anterior) and TS (triceps surae) muscles during PF (plantar-flexion) and DF (dorsi-flexion) MVCs (maximal voluntary contraction) in eccentric, isometric and concentric muscle contraction type.

		Coactivation level (%)	Mechanical ratio (%)
TA during PF MVC	Eccentric	7.3 ± 1.2^†^	2.0 ± 1.0 ***,^†^
	Isometric	9.8 ± 2.3	4.8 ± 3.7 ***
	Concentric	8.2 ± 1.1	3.1 ± 2.2 ***
TS during DF MVC	Eccentric	14.3 ± 3.0	15.9 ± 5.9^†^
	Isometric	12.6 ± 3.2	19.6 ± 3.4 ***
	Concentric	14.9 ± 5.7	11.6 ± 3.5 *^,†††,¥^

*p < 0.05, ***p < 0.001, significant difference between coactivation level and mechanical ratio.

^†^*p* < .0.05, ^†††^*p* < .0.001 significant difference with isometric condition.

^¥^*p* < .0.01, significant difference with eccentric condition.

### DF MVC

**Figure 2:**
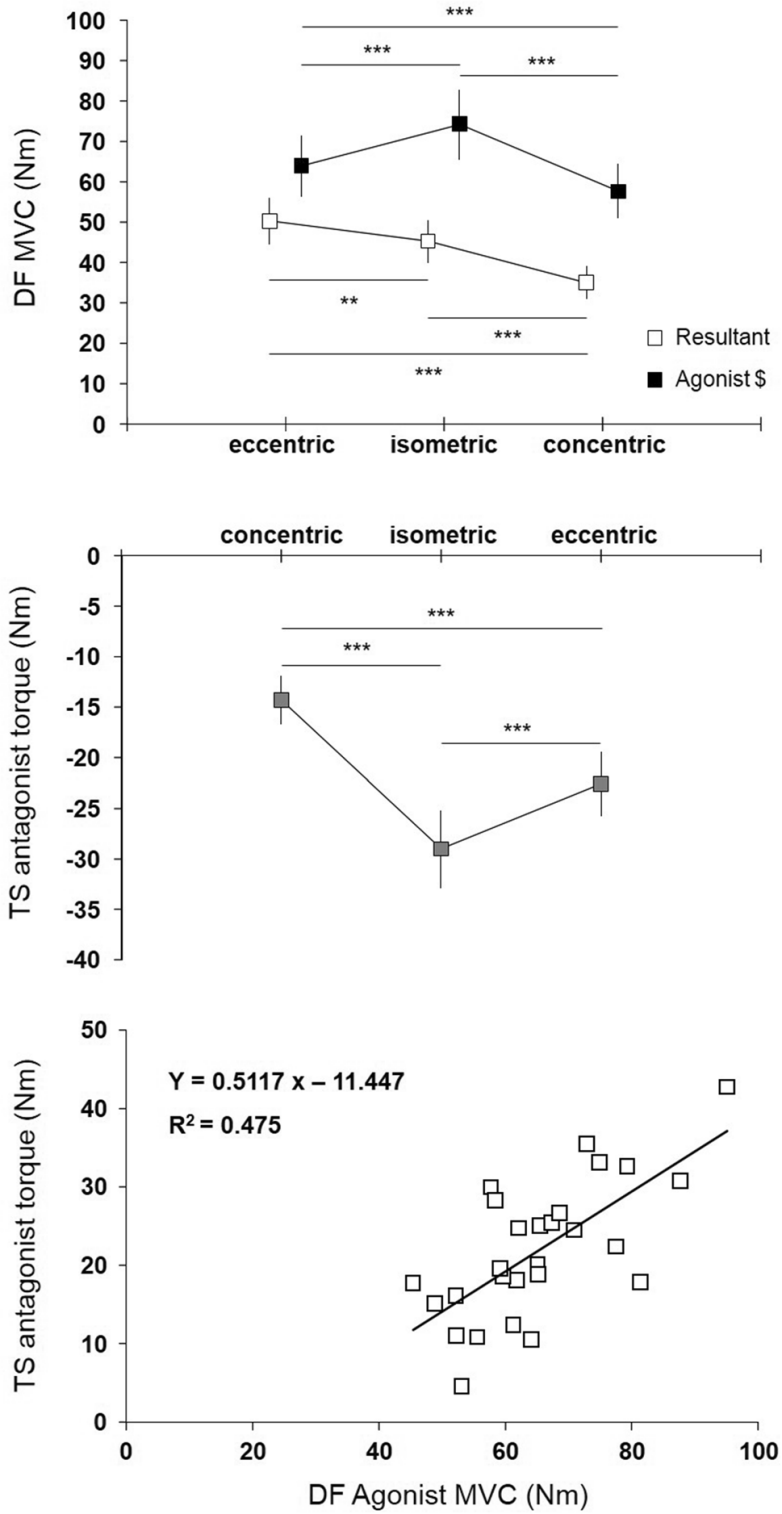
Mean DF resultant and agonist MVC (upper panel), mean TS antagonist torque (middle panel), relationship between agonist MVC and antagonist MVC in every muscle contraction type (lower panel).

Resultant and agonist MVC, TS antagonist torque. The two-way ANOVA analysis showed a significant main effect of considered DF MVC (resultant vs agonist MVC, *p* < 0.001, F = 117.05; d =  − 2.09 [− 2.77; − 1.41]), muscle contraction type (eccentric vs isometric vs concentric, *p* < 0.001, F = 40.219), and a significant interaction between MVC measurements and muscle contraction type (*p* < 0.001, F = 63.01) (Fig. 2). DF resultant MVC was significantly lower than DF agonist MVC for isometric (29.0 Nm, *p* < 0.001; d =  − 3.26 [− 4.79; − 1.74]), concentric (22.6 Nm, *p* < 0.001; d =  − 3.14 [− 4.64; − 1.65]) and eccentric (13.7 Nm, *p* = 0.001; d =  − 1.59 [− 2.74; − 0.45]) muscle contraction type. Considering DF resultant MVC, results showed that DF eccentric MVC (50.3 ± 6.7 Nm) was significantly higher than isometric (45.3 ± 6.1 Nm, *p* = 0.009; d = 0.77 [− 0.27; 1.81]) and concentric MVC (35.0 ± 5.5 Nm, *p* < 0.001; d = 2.48 [1.15; 3.81]), and isometric was significantly higher than concentric MVC (*p* < 0.001; d = 1.77 [0.59; 2.95]). Considering DF agonist MVC, results showed that DF isometric MVC (74.3 ± 10.2 Nm) was significantly higher than eccentric (64.0 ± 10.2 Nm, *p* < 0.001; d = 0.98 [− 0.08; 2.04]) and concentric MVC (57.6 ± 8.5 Nm, *p* < 0.001; d = 1.70 [0.53; 2.87]), and eccentric was significantly higher than concentric MVC (*p* < 0.001; d = 0.68 [− 0.35; 1.70]).

The one-way ANOVA showed significant main effect of muscle contraction type for the TS antagonist torque (*p* < 0.001, F = 60.05) (Fig. 2, middle panel). Post hoc analysis showed that the isometric TS antagonist torque was greater (29.0 ± 7.2 Nm) than eccentric TS antagonist torque (22.6 ± 6.6 Nm, *p* < 0.001; d = 0.93 [− 0.12; 1.98]) and concentric TS antagonist torque (14.3 ± 5.8 Nm, *p* < 0.001; d = 2.25 [0.98; 3.53]). A significant positive linear correlation was found between DF agonist MVC and TS antagonist torque (r = 0.69, *p* < 0.001) (Fig. 2, lower panel).

Eccentric/concentric ratio for TA resultant and agonist MVC. Statistical analysis showed that eccentric/concentric ratio was significantly higher for resultant (1.45 ± 0.13) than for agonist MVC (1.12 ± 0.15, *p* < 0.001, t = 9.25; d = 2.35 [1.05; 3.65]).

TS coactivation level versus mechanical ratio. Results showed no significant main effect of muscle contraction type (*p* = 0.54, F = 3.52) and coactivation method (*p* = 0.15, F = 2.55), whereas interaction between muscle contraction type and coactivation method was significant (*p* < 0.001, F = 16.21) (Table 2). The coactivation level was not significantly different between isometric, concentric, and eccentric muscle contraction types (*p* > 0.10). However, the mechanical ratio was significantly higher in the isometric than concentric (*p* < 0.001; d = 2.32 [1.02; 3.60]) and eccentric (*p* = 0.013; d = 0.76 [− 0.27; 1.80]) muscle contraction types. The mechanical ratio was significantly higher for eccentric compared to concentric muscle contraction type (*p* = 0.005; d = 0.88 [− 0.16; 1.93]). Post-hoc analysis showed that coactivation level was significantly lower than mechanical ratio in isometric condition (*p* < 0.001, t = 4.59; d =  − 2.14 [− 3.39; − 0.89]), while coactivation level was significantly higher than mechanical ratio in eccentric condition (*p* = 0.42, t = 2.16). No significant difference between coactivation method was found in the eccentric condition (*p* = 0.30, t = 1.07).

## Discussion

The current study investigated the mechanical impact of antagonist muscles related to their muscle contraction type during PF and DF MVC. In accordance with our hypothesis, results showed that resultant torque significantly underestimated agonist torque, with a major impact in DF MVC and a minor impact in PF MVC. In addition, TS antagonist torque depends on muscle contraction type resulting in DF MVC torque-velocity shape modification, while TA antagonist torque had no effect on PF MVC torque-velocity shape. The difference between DF eccentric and concentric MVC was attenuated considering agonist torque (12%) rather than resultant torque (45%).

In accordance with previous studies comparing resultant and agonist MVC in isometric condition^5,7,11^, the current study showed the overwhelming impact of PF muscles on DF resultant MVC (from 30 to 65%) and the minor impact of DF muscles on resultant PF MVC (~ 3%). Similar results were found in concentric and eccentric muscle contraction types, showing that the impact of antagonist torque on resultant MVC is highly specific as regards the muscles involved. It is safe to assume that the mechanical contribution of the DF muscles could be neglected when assessing PF performances^5,7,11^, while the impact of PF muscles should be quantified so as to provide an adequate reflection of DF strength capacity. Besides, even though our study emphasizes the crucial need to quantify agonist MVC, it bears mentioning that the resultant torque captures relevant and complementary information by reflecting the effective functional output of the neuromuscular system. We thereby recommend investigating both resultant and agonist MVC when considering maximal performance in strength and rehabilitation programs^21^.

Given this context, it matters to quantify the mechanical contribution of the coactivation phenomenon. By contrasting the antagonist torque estimation methods of the current bio-feedback with the most commonly used coactivation level, results showed that the ratio achieved by EMG activity significantly overestimated the impact of the antagonist muscle during PF MVC^5^. Furthermore, the coactivation level significantly underestimated the antagonist impact of the PF muscles during the DF isometric MVC, whereas it was overestimated in DF concentric MVC. All in all, our findings reinforce the hypothesis that the coactivation level cannot conclusively determine the mechanical contribution of the antagonist muscles in either DF or PF MVC^5,7,8,11^, especially in anisometric condition. As regards to the resultant MVC, the coactivation level should still be considered since it provides relevant and complementary information, helping to identify motor control alteration of electromechanical efficiency. Thereby, we suggest using both coactivation level and mechanical ratio depending on the issue addressed.

Focusing on PF muscles, our results did not highlight any differences between the coactivation level in isometric, concentric and eccentric muscle contraction type. Although these results corroborate the literature^12–16^, the antagonist torque was significantly more impacted by muscle contraction type. Our results indeed showed that the estimated TS antagonist torque was significantly greater in isometric than eccentric and concentric contractions. As a consequence, we observed that DF isometric agonist MVC became higher than DF eccentric and concentric agonist MVC (+ 16.5% and + 22%, respectively), while resultant torque showed greater DF eccentric MVC than isometric and concentric muscle contraction type. The shape of the ‘DF MVC-angular velocity’ relationship was thereby modified, highlighting less difference between eccentric and concentric MVC when considering agonist (12%) rather than the resultant (45%) MVC. In echo to the force–velocity relation of a muscle or a single fiber, in vitro studies have reported higher force during lengthening (eccentric) than shortening (concentric) or static (isometric) muscle contraction type^22,23^. While in vitro studies have reported that lengthening contraction force represents 1.4–1.9 times more than isometric^23–26^, in situ studies have reported similar^27^ and slight increases^15,16,20,28–31^ or no change^13,17,32–35^ in eccentric compared with isometric MVC. The shape of the force–velocity relation depends only on muscular factors in isolated fibers, whereas both neural and muscular components contribute to MVC in vivo. In addition, the specific control of muscles during lengthening is a subject of debate in the literature^18,19^. In a recent review, Duchateau and Enoka^19^ identified three points characterizing neural specificities during eccentric contractions. The authors reported that (i) EMG activity is most of the time lower in eccentric than in concentric effort^36–40^; (ii) neural activation is most of the time lower in eccentric than in concentric contraction^13,32,41^; (iii) peak discharge rate of motor units is lower in eccentric than in concentric contraction^42^. Studies have paradoxically reported an alteration of neural component and greater resultant MVC in eccentric compared to concentric contraction. Titin’s mechanical resistance mechanism described in the enhanced force in eccentric contractions may be still greater than the deficit of neural efficiency in eccentric compared to concentric contractions^43,44^. It could therefore be suggested that the greater PF eccentric resultant MVC observed in situ is partially due to the smaller antagonist torque in concentric, while high antagonist isometric and eccentric torques greatly impact isometric and concentric resultant MVC. Electromechanical efficacy, i.e. the ratio between EMG activity and the mechanical output, should be considered as a means of evaluating antagonist coactivation.

The quantification of the mechanical contribution of each muscle group in torque production about a specific joint may highlight the deficits between agonist and antagonist torques in athletes or in different pathologies. Practically speaking, assessment of the mechanical contribution of antagonist torque in isometric and anisometric conditions provides new opportunities to investigate antagonist muscles in injured athletes, for example by monitoring the antagonist/agonist ratio in hamstring as preventive measure or after injury^45,46^. Furthermore, this approach could improve understanding of the mechanical output synergies in muscles of patients with neurological disorders such as post-stroke patients^21,47^ by tailoring agonist and/or antagonist rehabilitation strength programs^21,47^ in addition to delivering medical therapy, such as toxin botulinum injection^48^ or ankle foot orthosis / functional electrical stimulation^49^ for gait recovery and quality of life improvement^50,51^.

### Methodological considerations

One of the main limitations in considering EMG activity for quantification of antagonist torque is the potential contamination of electrical signal captured from nearby muscles, known as the cross-talk phenomenon^52^. By using stimulation procedure to determine cross-talk^5,53^, we previously reported cross-talk lower than 11% for the TA and 8% for the TS^5^ as similarly reported in other studies^11,53,54^. More recently, Raiteri et al.^55^ investigated lengthening when ankle muscles acted as antagonists. While they claimed that co-contraction was negligible and largely reflected cross-talk activity in isometric conditions, muscle lengthening of TA, MG, LG and SOL ranging from 16.6 to 56.0% of the length changes were observed when muscles acted as agonists. Cross-talk might consequently over-estimate antagonist torque estimation.

Isometric and anisometric antagonist torque estimations were performed under sub-maximal level corresponding to a targeted EMG activity^5,7,8,11^. While this method was easy to perform in isometric conditions in which only two sub-maximal contractions were needed, it was more challenging in anisometric conditions, particularly when a low level of EMG activity was targeted. Given this context, 71% and 91% of data were retained for analysis in DF and PF sub-maximal contraction, respectively. Consequently, reliability measurement showed good to excellent reliability for TA antagonist torque (0.78 to 0.90), while excellent reliability was observed for TS antagonist torque (0.84 to 0.90). Our study indicated that the quantification of antagonist torque with the EMG biofeedback method is easy and reliable in anisometric conditions when medium and high levels of EMG activity are targeted (> 10%), but more challenging for a low level of EMG activity (< 10%).

In addition, our study provided results limited to a healthy male population at the ankle joint, while some previous studies reported that involved muscles^5,7–11^, age^8,11^, sex^9,10^, and pathologies^21,47^ could largely influence antagonist torque in isometric conditions. Future studies are needed to bring new insights in dynamic and more ecological conditions in different populations.

## Conclusion

Antagonist torque depends on muscle contraction type and strongly impacts DF resultant MVC in healthy men, while for PF, the relative contribution is small. In our study, antagonist torque was substantially altered by muscle contraction type affecting the ‘MVC-angular velocity’ relationship in DF, reducing the gap between eccentric and concentric MVC. In addition to the evaluation of the resultant MVC and the coactivation level, it matters when assessing muscular performance in isometric and anisometric contractions to consider the mechanical role of the antagonist muscles. Our findings provide new elements to assess performance and to better design strength training and rehabilitation programs related to agonist/antagonist capacity.

## Methods

### Participants

Nine (26.1 ± 2.7 years; 1.78 ± 0.05 m; 73.4 ± 6.5 kg) healthy men volunteered to participate in the 2 experimental sessions of the study. Subjects had no history of ankle surgery or other orthopaedic or neurological abnormalities of the lower limb during the two years preceding the study. The experimental design was approved by the regional ethics committee “CPP COOM III” and the French Agency for the Safety of Health Products “ANSM” (number: A00064-49), and was conducted in accordance with the principles of the Declaration of Helsinki for human experimentation. Written informed consent was obtained from all the study participants.

### Experimental protocol

After a short warm-up that consisted of two series of five sub-maximal voluntary contractions in each muscle contraction type, the subjects randomly performed two MVCs in isometric, eccentric and concentric conditions in PF and DF. If a variation of more than 5% occurred between the first and the second MVC, participants were asked to perform a third MVC. The trial resulting in the maximal torque developed was used for further analysis. During each maximal contraction, the subject was strongly encouraged by the experimenters and visual force feedback was provided. A two-min rest period was observed between each trial to avoid any fatiguing effect on the measurements.

Thereafter, participants were asked to randomly perform two sub-maximal isometric contractions in DF and PF, and ten eccentric and sub-maximal eccentric contractions corresponding to the target of an EMG activity displayed on a screen (please see ‘antagonist torque estimation’ section for details).

### Mechanical recording

Measurements were carried out in two experimental sessions by participants in order to examine reproducibility of antagonist torque measurement in isometric and anisometric conditions. Torque was measured using a dynamometer (System 3; Biodex Medical Systems, Shirley, NY). Subjects were seated with a knee angle of 120° (180° corresponds to full extension) and hip angle at 100°. The MVCs in DF and PF were measured in isometric conditions at 0° (perpendicular angle between the foot and the tibia), and in concentric (+ 10° s^-1^) and eccentric (− 10° s^-1^) conditions with a range of motion of 30° (i.e., − 15° DF and + 15° PF). To minimize trunk and hip movement during contraction, the waist was stabilized by means of a belt; arms were positioned across the chest. The right foot was attached to the dynamometer by means of the Biodex ankle attachment, which was customized with a shoe bolted to the foot plate at the heel. Standard toe straps were used over the shoe. The muscle contraction type (i.e., isometric, concentric or eccentric) and action (i.e., PF or DF) were randomized to avoid any systematic effects. In anisometric (i.e., concentric or eccentric) condition, the subjects were asked to pre-activate the agonist muscle by an isometric MVC 1-s before the ergometer movement.

### Electromyographic recording

EMG activity was concurrently measured with torque during maximal PF and DF efforts. EMG activity of the soleus, gastrocnemius medialis, gastrocnemius lateralis and Tibialis Anterior (TA) muscles was recorded by means of two silver–chloride surface electrodes of 10-mm diameter (Controle Graphique Medical, Brie-Comte-Robert, France), with an inter-electrode (center-to-center) distance of 25 mm. For the soleus, the recording electrodes were placed along the mid-dorsal line of the leg, three cm distal from the two heads of the gastrocnemius joining the Achilles tendon. Then, following the European recommendations regarding surface electromyography and the gastrocnemius medialis and lateralis^56^, electrodes were placed on the most prominent bulge of the muscle. The soleus and the gastrocnemius medialis and lateralis constitute the Triceps Surae (TS). In addition, electrodes were set at one third of the line between the tip of the fibula and the tip of the medial malleolus for the TA muscle. While other muscles were involved in DF (i.e., extensor digitorum longus, extensor halluces longus, and peroneus tertius), TA is considered as the prime mover representing the most powerful dorsal flexor muscle^5,57,58^. The ground electrode was attached on the patella of the other leg. Low impedance (< 5 kΩ) at the skin–electrode interface was obtained by shaving, abrading and cleaning the skin with an alcohol–ether–acetone mixture.

### Torque estimation

Antagonist torque estimation. Antagonist torque was estimated by an EMG biofeedback method specifically developed to assess antagonist torque^5,11^. Participants were asked to maintain a level of EMG activity according to the visual bio-feedback displayed on a screen: the current agonist EMG activity of the muscle had to correspond to its previously recorded antagonist EMG activity during the MVC^5^. For anisometric conditions, participants had to maintain EMG bio-feedback in isometric conditions during 2–3-s at − 15° or + 15°, depending on the action and muscle contraction type. Thereafter, the movement was started and the subjects were asked to maintain the targeted EMG activity level throughout the duration of the movement. For the EMG biofeedback task, the RMS value of the EMG signal was provided by an integrated circuit, true RMS-to-DC converter (model AD536A, Analog Devices, USA; characteristics: maximal error for true RMS-to-DC conversion = 0.5%, bandwidth > 450 kHz)^5,7,8,11^. For the TS muscles, this circuit instantaneously computed the true RMS level of the amplified EMG signal for the 3 channels separately with an integration time of 375 ms. In this way, the RMS of soleus, gastrocnemius lateralis and medialis muscles were calculated and summed up by the circuit. The latter value was then displayed on an oscilloscope as visual EMG bio-feedback^5,11^. Two isometric sub-maximal contractions were performed in isometric conditions with the target of the antagonist EMG activity recorded during isometric MVC; ten sub-maximal contractions were performed in concentric conditions corresponding to the antagonist EMG activity of the muscles acting in concentric conditions during the eccentric MVC; and ten sub-maximal contractions were performed in eccentric conditions corresponding to the antagonist EMG activity of the muscles acting in eccentric condition during the concentric MVC. Two contractions were sufficient to reach the EMG target in isometric condition, while 10 were performed to maintain EMG activity on the target during the movement in anisometric conditions.

#### Agonist torque estimation

Agonist torque is deduced by addition of the estimated antagonist torque to the recorded resultant torque depending on the muscle contraction type^5^:

1. For isometric contraction type: Agonist isometric MVC = Measured resultant isometric MVC + Antagonist isometric torque
2. For concentric contraction: Agonist concentric MVC = Measured concentric MVC + Antagonist eccentric torque
3. For eccentric contraction type: Agonist eccentric MVC = Measured eccentric MVC + Antagonist concentric torque

### Data analysis

Torque and EMG signals were acquired with a sampling frequency of 2 kHz and processed with a multi-channel analogue–digital converter (Biopac Systems Inc., USA). The EMG signal was filtered with bandwidth frequency ranging from 10 Hz to 5 kHz, (gain of 1000). EMG-RMS was measured over a 0.5-s period after the torque had reached a plateau for isometric conditions and a 0.5-s period divided into two periods of 0.25-s on both sides of the 90° position time in anisometric condition (i.e., constant angle of 2.5° under and over the 90° position) via Matlab software. For the anisometric condition, trials within 10% of the targeted EMG were kept for analysis; 91% and 71% of the trials were kept for analysis in PF and DF submaximal contraction, respectively. The ratio between eccentric and concentric MVC was calculated to compare MVC in anisometric muscle contraction type for the resultant and agonist MVCs. The level of coactivation corresponds to normalization of the EMG of a muscle acting as antagonist by the EMG activity of the same muscle maximally acting as agonist in a specific muscle contraction type^5,20^. In addition, we calculated a mechanical ratio corresponding to the antagonist torque normalized by the maximal agonist torque of the same muscle in a specific muscle contraction type^5^.

### Statistical analysis

All statistical tests were performed with Sigma Stat software (Sigma Stat 3.5, SPSS Inc., USA). Descriptive statistical methods, including means and their standard deviations (SDs), were calculated for each parameter. The data are presented as means ± SD in the text, the figures, and the table. Normality of the data was checked using the Shapiro–Wilk test, and equality of variances was verified by the Levene test. The reliability of our measurement was assessed with the Intra-Class Correlation (ICC), by performing a two-way random effects model with single measure reliability in which variance over the sessions 1 and 2 was considered^59^. For this study, ICC values from 0.60 to 0.79 were considered as “good reliability”, and those greater than 0.80 as “excellent reliability”^60,61^. In case of excellent or good reliability (Reproducibility: Intra-Class Correlation results), data were pooled, analyzed and presented as a mean of the 2 sessions. The coefficient of variation (CV) was calculated between the first and second session (i.e., the standard deviation divided by the mean). The statistical analyses were performed separately for PF and DF MVC, or TS and TA muscles. A two-way ANOVA with repeated measures was performed to examine the effect of the MVC (resultant vs. agonist), and muscle contraction type (eccentric, isometric, concentric) on PF and DF MVC. A one-way ANOVA with repeated measures was performed to assess the effect of muscle contraction type (eccentric, isometric, concentric) on TS and TA antagonist torque. A two-way ANOVA with repeated measures was performed to assess coactivation method (coactivation level vs mechanical ratio), and muscle contraction type (eccentric, isometric, concentric) on PF and DF MVC. Regardless of the muscle contraction type, Pearson correlation between agonist MVC and antagonist torque was performed. A Student T-test was performed to assess the eccentric/concentric MVC ratio between resultant and agonist MVC. When a significant main effect was found, Tukey post hoc test was performed to identify the significant differences between factors. The standardized effect size was calculated using Cohen d for paired samples, adjusted for the correlation between condition and using the Hedge bias correction^62^. The level of significance was set at *p* < 0.05.

## Data Availability

The datasets generated during and/or analysed during the current study are available from the corresponding author on reasonable request.

## References

[1] Solomonow M, Baratta R, Zhou BH, D’Ambrosia R (1988). Electromyogram coactivation patterns of the elbow antagonist muscles during slow isokinetic movement. Exp. Neurol..

[2] Kellis E (1998). Quantification of quadriceps and hamstring antagonist activity. Sports Med..

[3] Remaud A, Guével A, Cornu C (2007). Antagonist muscle coactivation and muscle inhibition: Effects on external torque regulation and resistance training-induced adaptations. Neurophysiol. Clin..

[4] Falconer K, Winter DA (1985). Quantitative assessment of co-contraction at the ankle joint in walking. Electromyogr. Clin. Neurophysiol..

[5] Billot M, Simoneau E, Van Hoecke J, Martin A (2010). Coactivation at the ankle joint is not sufficient to estimate agonist and antagonist mechanical contribution. Muscle Nerve.

[6] Nielsen JB (1998). Co-contraction of antagonistic muscles in man. Dan. Med. Bull..

[7] Billot M, Simoneau EM, Ballay Y, Van Hoecke J, Martin A (2011). How the ankle joint angle alters the antagonist and agonist torques during maximal efforts in dorsi- and plantar flexion. Scand. J. Med. Sci. Sports.

[8] Billot M, Duclay J, Simoneau-Buessinger EM, Ballay Y, Martin A (2014). Is co-contraction responsible for the decline in maximal knee joint torque in older males?. Age (Dordr.).

[9] Krishnan C, Williams GN (2010). Error associated with antagonist muscle activity in isometric knee strength testing. Eur. J. Appl. Physiol..

[10] Krishnan C, Williams GN (2009). Sex differences in quadriceps and hamstrings EMG-moment relationships. Med. Sci. Sports Exerc..

[11] Simoneau EM, Billot M, Martin A, Van Hoecke J (2009). Antagonist mechanical contribution to resultant maximal torque at the ankle joint in young and older men. J. Electromyogr. Kinesiol..

[12] Abbruzzese G, Morena M, Spadavecchia L, Schieppati M (1994). Response of arm flexor muscles to magnetic and electrical brain stimulation during shortening and lengthening tasks in man. J. Physiol..

[13] Amiridis IG (1996). Co-activation and tension-regulating phenomena during isokinetic knee extension in sedentary and highly skilled humans. Eur. J. Appl. Physiol. Occup. Physiol..

[14] Barrué-Belou S, Amarantini D, Marque P, Duclay J (2016). Neural adaptations to submaximal isokinetic eccentric strength training. Eur. J. Appl. Physiol..

[15] Duclay J, Pasquet B, Martin A, Duchateau J (2011). Specific modulation of corticospinal and spinal excitabilities during maximal voluntary isometric, shortening and lengthening contractions in synergist muscles. J. Physiol..

[16] Pasquet B, Carpentier A, Duchateau J (2006). Specific modulation of motor unit discharge for a similar change in fascicle length during shortening and lengthening contractions in humans. J. Physiol..

[17] Babault N, Pousson M, Ballay Y, Van Hoecke J (2001). Activation of human quadriceps femoris during isometric, concentric, and eccentric contractions. J. Appl. Physiol..

[18] Duchateau J, Baudry S (2014). Insights into the neural control of eccentric contractions. J. Appl. Physiol..

[19] Duchateau J, Enoka RM (2016). Neural control of lengthening contractions. J. Exp. Biol..

[20] Kellis E, Baltzopoulos V (1998). Muscle activation differences between eccentric and concentric isokinetic exercise. Med. Sci. Sports Exerc..

[21] Palmer JA, Zarzycki R, Morton SM, Kesar TM, Binder-Macleod SA (2017). Characterizing differential poststroke corticomotor drive to the dorsi- and plantarflexor muscles during resting and volitional muscle activation. J. Neurophysiol..

[22] Abbott BC, Aubert XM (1952). The force exerted by active striated muscle during and after change of length. J. Physiol..

[23] Katz B (1939). The relation between force and speed in muscular contraction. J Physiol.

[24] Deleze JB (1961). The mechanical properties of the semitendinosus muscle at lengths greater than its length in the body. J. Physiol..

[25] Edman KA (1988). Double-hyperbolic force-velocity relation in frog muscle fibres. J. Physiol..

[26] Hill AV (1964). The effect of load on the heat of shortening of muscle. Proc. R. Soc. Lond. B Biol. Sci..

[27] Hahn D (2018). Stretching the limits of maximal voluntary eccentric force production in vivo. J. Sport Health Sci..

[28] Aagaard P (2000). Neural inhibition during maximal eccentric and concentric quadriceps contraction: effects of resistance training. J Appl. Physiol..

[29] Linnamo V, Strojnik V, Komi PV (2006). Maximal force during eccentric and isometric actions at different elbow angles. Eur. J. Appl. Physiol..

[30] Reeves ND, Narici MV (2003). Behavior of human muscle fascicles during shortening and lengthening contractions in vivo. J. Appl. Physiol..

[31] Hahn D, Hoffman BW, Carroll TJ, Cresswell AG (2012). Cortical and spinal excitability during and after lengthening contractions of the human plantar flexor muscles performed with maximal voluntary effort. PLoS ONE.

[32] Beltman JGM, Sargeant AJ, van Mechelen W, de Haan A (2004). Voluntary activation level and muscle fiber recruitment of human quadriceps during lengthening contractions. J. Appl. Physiol..

[33] Colson S, Pousson M, Martin A, Van Hoecke J (1999). Isokinetic elbow flexion and coactivation following eccentric training. J. Electromyogr. Kinesiol..

[34] Pinniger GJ, Steele JR, Thorstensson A, Cresswell AG (2000). Tension regulation during lengthening and shortening actions of the human soleus muscle. Eur. J. Appl. Physiol..

[35] Westing SH, Cresswell AG, Thorstensson A (1991). Muscle activation during maximal voluntary eccentric and concentric knee extension. Eur. J. Appl. Physiol. Occup. Physiol..

[36] Altenburg TM, de Ruiter CJ, Verdijk PWL, van Mechelen W, de Haan A (2009). Vastus lateralis surface and single motor unit electromyography during shortening, lengthening and isometric contractions corrected for mode-dependent differences in force-generating capacity. Acta Physiol. (Oxf.).

[37] Duclay J, Martin A (2005). Evoked H-reflex and V-wave responses during maximal isometric, concentric, and eccentric muscle contraction. J. Neurophysiol..

[38] Garner JC, Blackburn T, Weimar W, Campbell B (2008). Comparison of electromyographic activity during eccentrically versus concentrically loaded isometric contractions. J. Electromyogr. Kinesiol..

[39] Linnamo V, Bottas R, Komi PV (2000). Force and EMG power spectrum during and after eccentric and concentric fatigue. J. Electromyogr. Kinesiol..

[40] Linnamo V, Moritani T, Nicol C, Komi PV (2003). Motor unit activation patterns during isometric, concentric and eccentric actions at different force levels. J. Electromyogr. Kinesiol..

[41] Westing SH, Seger JY, Thorstensson A (1990). Effects of electrical stimulation on eccentric and concentric torque-velocity relationships during knee extension in man. Acta Physiol. Scand..

[42] Del Valle A, Thomas CK (2005). Firing rates of motor units during strong dynamic contractions. Muscle Nerve.

[43] Herzog W (2014). Mechanisms of enhanced force production in lengthening (eccentric) muscle contractions. J. Appl. Physiol..

[44] Herzog W, Lee EJ, Rassier DE (2006). Residual force enhancement in skeletal muscle. J. Physiol..

[45] Avrillon S, Hug F, Guilhem G (2020). Bilateral differences in hamstring coordination in previously injured elite athletes. J. Appl. Physiol..

[46] Opar DA, Williams MD, Shield AJ (2012). Hamstring strain injuries: Factors that lead to injury and re-injury. Sports Med..

[47] Klein CS, Brooks D, Richardson D, McIlroy WE, Bayley MT (2010). Voluntary activation failure contributes more to plantar flexor weakness than antagonist coactivation and muscle atrophy in chronic stroke survivors. J. Appl. Physiol..

[48] Gupta AD (2018). A systematic review: Efficacy of botulinum toxin in walking and quality of life in post-stroke lower limb spasticity. Syst. Rev..

[49] David R (2022). A 6-month home-based functional electrical stimulation program for foot drop in a post-stroke patient: Considerations on a time course analysis of walking performance. Int. J. Environ. Res. Public Health.

[50] Dobkin BH (2004). Strategies for stroke rehabilitation. Lancet Neurol..

[51] Dobkin BH (2005). Clinical practice. Rehabilitation after stroke. N. Engl. J. Med..

[52] Winter DA, Fuglevand AJ, Archer SE (1994). Crosstalk in surface electromyography: Theoretical and practical estimates. J. Electromyogr. Kinesiol..

[53] Solomonow M (1994). Surface and wire EMG crosstalk in neighbouring muscles. J. Electromyogr. Kinesiol..

[54] Carpentier A, Duchateau J, Hainaut K (1999). Load-dependent muscle strategy during plantarflexion in humans. J. Electromyogr. Kinesiol..

[55] Raiteri BJ, Cresswell AG, Lichtwark GA (2015). Ultrasound reveals negligible cocontraction during isometric plantar flexion and dorsiflexion despite the presence of antagonist electromyographic activity. J. Appl. Physiol..

[56] Hermens HJ, Freriks B, Disselhorst-Klug C, Rau G (2000). Development of recommendations for SEMG sensors and sensor placement procedures. J. Electromyogr. Kinesiol..

[57] Marsh E, Sale D, McComas AJ, Quinlan J (1981). Influence of joint position on ankle dorsiflexion in humans. J. Appl. Physiol. Respir. Environ. Exerc. Physiol..

[58] Fukunaga T, Roy RR, Shellock FG, Hodgson JA, Edgerton VR (1996). Specific tension of human plantar flexors and dorsiflexors. J. Appl. Physiol..

[59] Shrout PE, Fleiss JL (1979). Intraclass correlations: uses in assessing rater reliability. Psychol. Bull..

[60] Atkinson G, Nevill AM (1998). Statistical methods for assessing measurement error (reliability) in variables relevant to sports medicine. Sports Med..

[61] Morrow JR, Jackson AW (1993). How ‘significant’ is your reliability?. Res. Q Exerc. Sport.

[62] Cohen, J. *Statistical Power Analysis for the Behavioral Sciences*. (1988).

